# Eye-Closure Rate Modulation in Blepharospasm

**DOI:** 10.5334/tohm.748

**Published:** 2023-08-24

**Authors:** Panagiotis Kassavetis, Ejaz A. Shamim, Kranz Gottfried, Mark Hallett

**Affiliations:** 1 Department of Neurology, University of Utah, Salt Lake City, UT, USA; 2 National Institute of Neurological Disorders and Stroke, National Institutes of Health, Bethesda, MD, USA; 3 Mid-Atlantic Permanent Research Institute, Mid-Atlantic Permanente Medical Group, Kaiser Permanente, Mid-Atlantic States, Rockville, MD 20854, USA; 4 Department of Neurological Rehabilitation, Clinic for Rehabilitation Rosenhügel, Vienna, Austria

**Keywords:** Blepharospasm, dystonia, eye-closure, relatives

## Abstract

**Background:**

Blepharospasm (BSP) is a type of focal dystonia and a number of patients with BSP have relatives also affected by BSP. The objective of this study was to quantify eye closure rates during activities of daily living in individuals with BSP and individuals without BSP with and without a first-degree relative with BSP.

**Methods:**

37 patients with BSP (BSP group), 10 asymptomatic volunteers with a first-degree relative with BSP (RELATIVES group) and 25 asymptomatic volunteers without relatives with BSP (HV group) were recruited. The number of eye closures for each task were counted per 60 seconds, with a video recording. Within and between groups statistical comparisons of eye-closure rates were performed.

**Results:**

The eye-closure rates of the RELATIVES group were not different from the BSP group for the majority of the tasks (except for watching television), and the HV group (for all tasks). The rate of eye closures in the BSP group compared to HV, was significantly increased in two tasks, resting and watching television.

**Discussion:**

Eye closure rate varies considerably during activities of daily living in all groups. Individuals with first degree relative with BSP are more likely to have increased eye closure rate at rest.

## Introduction

Blepharospasm (BSP), a type of focal dystonia (FD), affects the periorbital muscles causing increased blinking frequency and ocular spasms preventing the eyes from opening. BSP has a heterogeneous etiology and isolated BSP is usually idiopathic although hereditary factors have been described but not precisely identified [1,2]. A characteristic feature of BSP and other FDs is the fluctuation of symptoms dependent on the task that is being performed. For example, during reading the blink rate has been found to be significantly lower compared to staring with eyes in the primary position in patients with BSP, which is similar to blink rate modulation in healthy volunteers [3,4]. Another task that may lower the blink rate is speaking, although this has not been replicated [5,6].

Currently, there is no test to definitively diagnose BSP and the gold standard is a clinical diagnosis based on history, clinical features, and phenomenology [7,8,9]. For that reason, a detailed description of BSP symptoms is important. The natural variability of BSP can be confused with a functional disorder. In fact, originally BSP and other FD were thought to be functional [10]. Therefore, good understanding of the modulation of the BSP symptoms during different daily activities can be very helpful clinically.

The improvement of symptoms with sensory tricks is another feature of BSP and other FDs [11]. The physiology of sensory tricks is not clear but it is possible that neural networks involved in sensory tricks are also active during certain motor tasks leading to an improvement in BSP symptoms. We hypothesize that patients who have sensory tricks may show a differential modulation of the symptoms depending on the motor tasks they are performing.

In this study, we investigated the modulation of eye closure rates during several activities of daily living in participants with and without BSP. In addition, we tested a group of asymptomatic individuals who have a first-degree relative with BSP. We also tested the hypothesis that the presence of sensory tricks can predict modulation of BSP symptoms.

## Methods

### Participants

Three groups of participants were recruited during a local patient group meeting in Maryland, USA: 37 patients with BSP **(BSP group)** (29 females, mean age 62.5 years, SD 8.65), 10 asymptomatic healthy volunteers who have a first-degree relative (mother, father, sibling, and/or children) with BSP **(RELATIVES group)** (4 females, mean age 53.4 years, SD 19.9) and 25 age-matched healthy volunteers (HVs) who have no first-degree relatives with BSP **(HV group)** (14 females, mean age 59.6 years, SD 9.06). This was the total number of participants available for recruitment.

Of the 37 patients with BSP, 22 had pure BSP, 11 had involvement of the lower face (cranial dystonia, Meige syndrome) and 4 also had involvement of the neck region (cranial-cervical dystonia, segmental dystonia). The average duration of the disease was 9.8 ± 7.4 years. Twenty-two out of 35 BSP patients (63%) reported the presence of a sensory trick (data on sensory trick are missing for 2 BSP patients). 35 received Botulinum toxin injections and 5 of them were also on oral medication (anticholinergic or muscle relaxant). The average timing of enrolment was 17.6 weeks (range 1–108) after the last Botulinum toxin injections.

The study was approved by the Institutional Review Board of the National Institute of Neurological Disorders and Stroke and conducted in accordance with the Declaration of Helsinki. All participants provided their written informed consent before participation.

### Design

A standardized video recording was obtained for all participants. During the video, each participant sat comfortably on a chair while a video camera was focused on their face. The participants performed 10 different tasks for at least 80 seconds per task. The first 20 seconds of each task were not analyzed in order to let the subjects adjust to every task. The tasks were: 1. Resting, 2. Talking about a neutral topic, 3. Reading a passage from a book, 4. Peeling an orange with their hands, 5. Eating an orange, 6. Counting out loud, ascending order, steps of one, starting at one, 7. Counting out loud, descending order, steps of seven, starting at one hundred (also known as serial 7s), 8. Typing, 9. Watching television, 10. Chewing gum. All the videos were stored for offline evaluation. Two investigators watched all the videos and counted the number of eye closures for each task. As blinks and spasms are sometimes difficult to separate, the measure in this study is formally that of the number of eye closures in 60 seconds. One eye closure was defined as a full closure of eyelids with the lids touching each other.

### Statistical analysis

The distribution of the eye closure data was evaluated with the Shapiro-Wilk test. Non-normal data were transformed using the formula y = Log10(x+1), where y is the transformed value and x is the original value. Adding 1 to every original value allows Log10 transformation of all values including those equal to 0. Data distribution after transformation was tested again with the Shapiro-Wilk test. Sphericity was evaluated with Mauchly’s test and equality of variance was tested with Levene’s test.

Repeated measured ANOVA (rmANOVA) with within-subject factor TASK (10 levels; one level for each task) and between-subjects factor GROUP (3 levels: BSP vs RELATIVES vs HV) was performed to compare eye closures between tasks, between groups, and their interaction. Post hoc t-tests were used to further investigate differences in eye closures between groups for each task, with Bonferroni correction (all reported p-values are corrected with Bonferroni correction for multiple comparisons). In order to compare the number of eye closures between patients with and without sensory trick we used rmANOVA with within-subject factor TASK (10 levels; one level for each task) and between-subjects factor TRICK (2 levels: with vs without sensory trick). Participants with missing data were not included in the analysis. In order to compare the effect of gender on eye closure rate, an independent sample t-test was used for parametric data and Mann-Whitney test for non-parametric data for each task.

## Results

**Figure 1:**
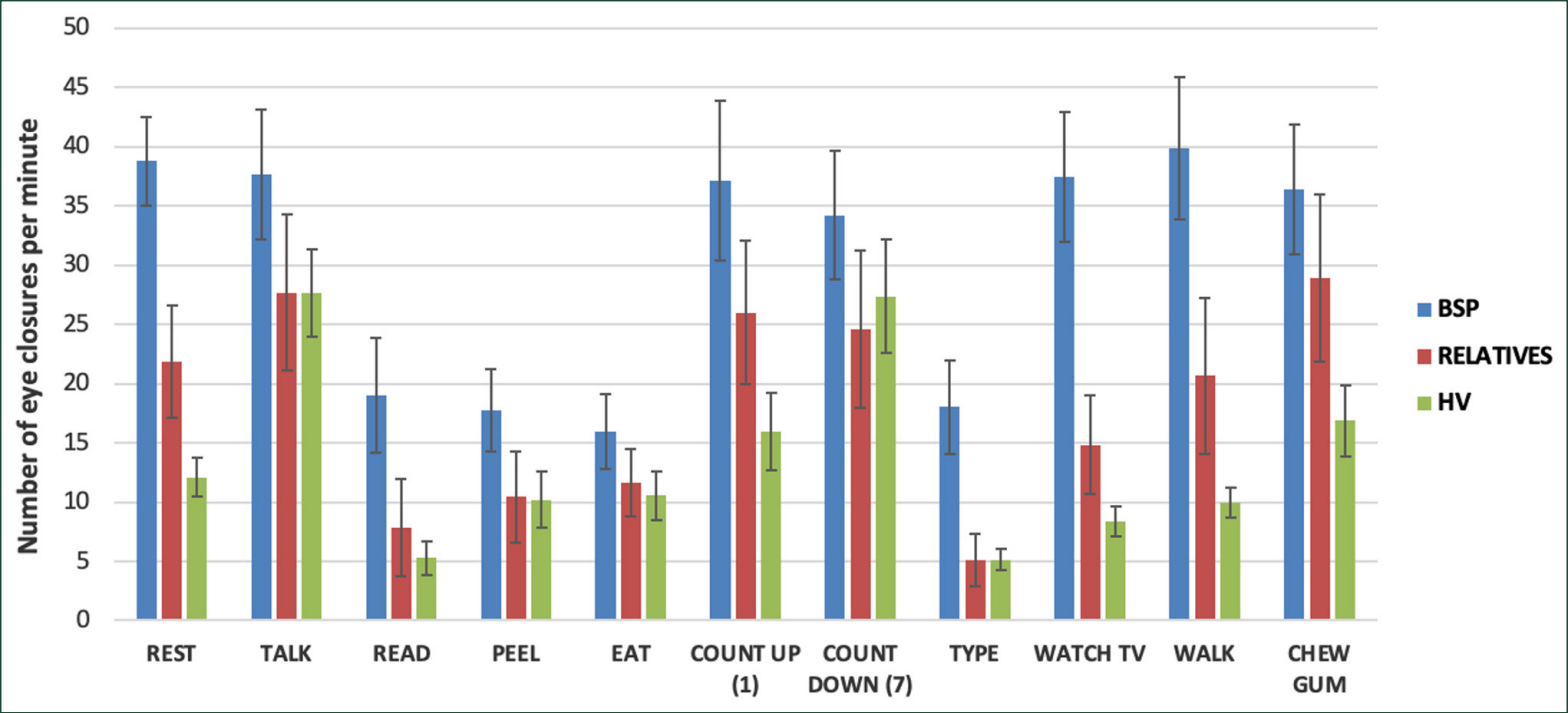
Eye closure rates during 10 tasks in BSP, Relatives and HV groups. Error bars represent standard error of the mean.

rmANOVA showed significant effect of TASK (F (9,558) = 33.72, p < 0.01), significant effect of GROUP (F(2,62) = 4.62, p = 0.01) and significant interaction TASKxGROUP (F(18,558) = 3.58, p < 0.01) (Figure 1).

In order to explore the effects of factor GROUP (irrespective of TASK), post hoc pair-wise comparisons with Bonferroni correction (3 comparisons), showed that the BSP group had more eye closure counts compared to the HV group (t(0.05, 0.52) = 3.01, p = 0.01) but not different compared to the RELATIVES group (t(–0.17, 0.50) = 1.20, p = 0.7. The HV group was not different from the RELATIVES group t(–0.23,0.48) = 0.86, p = 1.0.

In order to explore the effects of factor TASK (irrespectively of GROUP), post hoc pair-wise comparisons with Bonferroni correction (45 comparisons) showed that eye closure rate was lower in four tasks compared to resting (reading t(0.33,0.73) = 8.71, p < 0.01, pealing an orange t(0.24,0.64) = 7.19, p < 0.01, eating an orange t(0.090,0.48) = 4.76, p < 0.01 and typing t(0.35, 0.75) = 9.00, p < 0.01).

In order to explore the interaction TASKxGROUP, one-way ANOVA for each task (Bonferroni correction for 10 ANOVAs), showed a significant difference in eye closure counts among groups for the tasks: resting (F (2,69) = 17.88, p < 0.01) and watching television (F(2,68) = 18.37, p < 0.01). For the resting task, post-hoc pair-wise comparisons between the groups (3 comparisons) showed that the HV group had significantly lower eye closure counts compared to the BSP group (t(0.33, 0.66) = 6.09, p < 0.01), whereas the RELATIVE group was not different compared to the HV or the BSP group. For the watching television task post-hoc pair-wise comparisons between the groups (3 comparisons) showed that the HV group had significantly smaller eye closure counts compared to the BSP group (t(0.35, 0.82) = 5.89, p < 0.01), whereas the RELATIVE group had significantly smaller eye closure counts compared to the BSP group (t(0.10, 0.75) = 3.11, p < 0.01) but there was no significant difference between the HV and BSP groups.

Sensory trick analysis with rmANOVA with within-subject factor TASK (10 levels; one level for each task) and between-subjects factor TRICK (2 levels: with vs without sensory trick) showed significant effect of factor TASK (F (9,297) = 17.98, p < 0.001) and non-significant effect of factor TRICK (F (1,33) = 0.20, p = 0.66) or their interaction TASKxTRICK (F (9,297) = 0.33, p = 0.96).

There was no significant difference in the eye closure rates between genders for all the tasks (p > 0.05).

## Discussion

In this study, we examined the modulation of eye closure rate during 10 different tasks in a group of patients with BSP (BSP group), a group of asymptomatic volunteers who have a 1^st^-degree relative with BSP (RELATIVES group), and a group of asymptomatic volunteers without any first degree relative with BSP (HV group). The main findings of this study are: 1. The mean rate of eye closure was higher in the BSP group compared to the HV group, and for some tasks such as resting, reading, typing and watching television the difference was 3-fold. 2. Eye closure rates changed significantly during different motor tasks in all the groups. Even in the HV group, the mean eye closure rates ranged from 5 mean eye closures per 60 seconds during typing to 28 mean eye closures per 60 seconds during talking. 3. In the RELATIVES group the rate of eye closure was not significantly different from the HV or the BSP group (except for one task: watching television), and 4. In the BSP group, the presence of sensory trick did not have impact on the eye closure rate in different tasks.

This study highlights the fluctuations of the symptoms in patients with blepharospasm. Variability of the symptoms can indicate functional etiology for several movement disorders [12,13]. However, symptoms of non-functional movement disorders can also fluctuate naturally [14]. In this study, we show that the eye closure rate fluctuates significantly in patients with BSP and healthy volunteers, between tasks. However, this study did not investigate the variability of eye closure within the same tasks over time, which is a common feature of functional neurological disorders [15]. The baseline eye closure rates are consistent with prior literature [3,6,16]. In patients with BSP, the eye closure rate can almost be halved during certain tasks compared to rest. In particular, reading (which has been described before [3,6], peeling an orange, eating an orange, and typing were the tasks with the lowest eye closures rates for all the groups. Similar to Ferrazzano et al. 2019 [5], we did not find modulation of eye closure rate during talking (or the two counting tasks) in the BSP group, which is in contrast to previously reported results by Bentivoglio et al 2006 [6]. Notably, during motor tasks that involve the hands (typing and pealing an orange), the eye closure rate was reduced in the BSP group, similar to the previously reported effect of writing [5]. Reduction of symptoms during motor tasks that involve the hands can be falsely interpreted as distractibility, which is another indication of functional etiology for other movement disorders. In the HV group, we replicated previous results that the eye closure rate increases during talking [6]. Regarding the role of sensory trick, we could not prove our hypothesis that the presence of sensory trick in some BSP patients could predict the modulation of symptoms during different tasks.

This is the first time that first-degree relatives of patients with BSP were systematically examined during several motor tasks. The lack of significant difference does not prove equality but we cannot ignore the fact that the mean eye closure rate in this group was between the means of the BSP and HV groups for most tasks. Statistical comparisons showed that the eye closure rate in the RELATIVES group was not significantly different from the HV group or the BSP group (except for one task: watching television). It is possible that genetic pre-disposition in this group can explain a tendency towards higher eye closure rates that are not so high as to become clinically significant. Environmental factors could also play a role in this finding. However, this study does not provide any evidence to prove any genetic or environmental effect. This result is similar to abnormal temporal discrimination thresholds in first-degree relatives of patients with sporadic adult-onset primary torsion dystonia [17].

Surprisingly, the eye closure rate was not different between the BSP group and HV group during most of the tasks (significant difference for only two tasks: resting and watching television). It is possible that the inclusion of the RELATIVES group in this study had a “blurring” statistical effect by adding more comparisons and increasing the threshold for statistical significance with a very conservative correction for multiple comparisons. Regardless, it is reassuring that resting was one of the tasks where a significant difference was found, as this is probably the most commonly tested task in the clinical setting.

A distinction between blink rates and spasms of the peri-orbital muscles is often attempted in phenotyping studies of BSP. However, in this study, our main variable was eye closure, without distinction between blinks and spasms. This is a potential limitation of this study. However, counting blinks and spasms can be challenging during a clinical visit, and in that respect, our results are more generalizable (outside of a research setting). The eye closure rate which was measured in this study is different from the blink rate because we included any type of eye closure in the measurements, including blinks and spasms. Increased blink rate alone has been used as a simple measure to assess response to treatment in blepharospasm [18] but it has low specificity and sensitivity for the diagnosis of BSP [9] and its pathophysiology may be different than orbicularis oculi muscle spasms [19]. Another limitation is the relatively low number of participants which may not be adequate for such a high number of comparisons. The design of the study with the use of a video recording can also be considered a limitation as in-person clinical evaluation is the gold standard for assessment of BSP. However, video recordings allow re-review for accurate counting of eye closures, and they are commonly used in similar studies [3,5,6]. Finally, the fact that some of the patients had received botulinum toxin injections less than 12 weeks prior to the data collection, might have caused an underestimation of the eye closure rate for some patients with BSP. However, there is no evidence that botulinum toxin can affect eye closure rate differentially during different tasks.

In conclusion, eye closure rates vary naturally during different motor tasks. Clinicians should be aware of this fluctuation to avoid mislabeling of BSP as functional solely based on variability. Clinical examination at rest is adequate to differentiate participants with and without BSP. First degree relatives of BSP may have higher eye closure rates, even if they are asymptomatic. The presence of sensory trick is not useful in predicting variability of symptoms during different tasks.

## References

[1] Defazio G, Livrea P. Primary blepharospasm: diagnosis and management. Drugs. 2004; 64(3): 237–44. DOI: 10.2165/00003495-200464030-00002 14871168

[2] Defazio G, Brancati F, Valente EM, Caputo V, Pizzuti A, Martino D, et al. Familial blepharospasm is inherited as an autosomal dominant trait and relates to a novel unassigned gene. Mov Disord. 2003; 18(2): 207–12. DOI: 10.1002/mds.10314 12539217

[3] Richard MJ, Woodward DJ, McCoy AN, Woodward JA. Effect of reading on surface electromyogram recordings in patients with blepharospasm. Ophthalmic Plast Reconstr Surg. 2009; 25(5): 378–81. DOI: 10.1097/IOP.0b013e3181b0d630 19966652

[4] Bentivoglio AR, Bressman SB, Cassetta E, Carretta D, Tonali P, Albanese A. Analysis of blink rate patterns in normal subjects. Mov Disord. 1997; 12(6): 1028–34. DOI: 10.1002/mds.870120629 9399231

[5] Ferrazzano G, Conte A, Belvisi D, Fabbrini A, Baione V, Berardelli A, et al. Writing, reading, and speaking in blepharospasm. J Neurol. 2019; 266(5): 1136–40. DOI: 10.1007/s00415-019-09243-x 30783748

[6] Bentivoglio AR, Daniele A, Albanese A, Tonali PA, Fasano A. Analysis of blink rate in patients with blepharospasm. Mov Disord. 2006; 21(8): 1225–9. DOI: 10.1002/mds.20889 16622858

[7] Defazio G, Hallett M, Jinnah HA, Berardelli A. Development and validation of a clinical guideline for diagnosing blepharospasm. Neurology. 2013; 81(3): 236–40. DOI: 10.1212/WNL.0b013e31829bfdf6 23771487 PMC3770163

[8] Colosimo C, Bologna M, Berardelli A. How Do I Examine Blepharospasm? Mov Disord Clin Pract. 2015; 2(4): 449. DOI: 10.1002/mdc3.12291 30838249 PMC6353484

[9] Defazio G, Jinnah HA, Berardelli A, Perlmutter JS, Berkmen GK, Berman BD, et al. Diagnostic criteria for blepharospasm: A multicenter international study. Parkinsonism Relat Disord. 2021; 91: 109–14. DOI: 10.1016/j.parkreldis.2021.09.004 34583301 PMC9048224

[10] Marsden CD. The problem of adult-onset idiopathic torsion dystonia and other isolated dyskinesias in adult life (including blepharospasm, oromandibular dystonia, dystonic writer’s cramp, and torticollis, or axial dystonia). Adv Neurol. 1976; 14: 259–76. 941774

[11] Pandey S, Soni G, Sarma N. Sensory tricks in primary blepharospasm and idiopathic cervical dystonia. Neurol India. 2017; 65(3): 532–6. DOI: 10.4103/neuroindia.NI_864_16 28488615

[12] Hallett M. Functional (psychogenic) movement disorders – Clinical presentations. Parkinsonism Relat Disord. 2016; 22(Suppl 1): S149–52. DOI: 10.1016/j.parkreldis.2015.08.036 26365778 PMC4662613

[13] Espay AJ, Aybek S, Carson A, Edwards MJ, Goldstein LH, Hallett M, et al. Current Concepts in Diagnosis and Treatment of Functional Neurological Disorders. JAMA Neurol. 2018; 75(9): 1132–41. DOI: 10.1001/jamaneurol.2018.1264 29868890 PMC7293766

[14] Berlot R, Rothwell JC, Bhatia KP, Kojovic M. Variability of Movement Disorders: The Influence of Sensation, Action, Cognition, and Emotions. Mov Disord. 2021; 36(3): 581–93. DOI: 10.1002/mds.28415 33332680

[15] Hallett M, Aybek S, Dworetzky BA, McWhirter L, Staab JP, Stone J. Functional neurological disorder: new subtypes and shared mechanisms. Lancet Neurol. 2022; 21(6): 537–50. DOI: 10.1016/S1474-4422(21)00422-1 35430029 PMC9107510

[16] Ranti C, Jones W, Klin A, Shultz S. Blink Rate Patterns Provide a Reliable Measure of Individual Engagement with Scene Content. Sci Rep. 2020; 10(1): 8267. DOI: 10.1038/s41598-020-64999-x 32427957 PMC7237680

[17] Kimmich O, Bradley D, Whelan R, Mulrooney N, Reilly RB, Hutchinson S, et al. Sporadic adult onset primary torsion dystonia is a genetic disorder by the temporal discrimination test. Brain. 2011; 134(Pt 9): 2656–63. DOI: 10.1093/brain/awr194 21840890

[18] Jang J, Lew H. Blink index as a response predictor of blepharospasm to botulinum neurotoxin-A treatment. Brain Behav. 2021; 11(11): e2374. DOI: 10.1002/brb3.2374 34555267 PMC8613441

[19] Conte A, Ferrazzano G, Defazio G, Fabbrini G, Hallett M, Berardelli A. Increased Blinking May Be a Precursor of Blepharospasm: A Longitudinal Study. Mov Disord Clin Pract. 2017; 4(5): 733–6. DOI: 10.1002/mdc3.12499 29082270 PMC5654574

